# Bioinspired Edible Lubricant-Infused Surface with Liquid Residue Reduction Properties

**DOI:** 10.34133/2019/1649427

**Published:** 2019-10-10

**Authors:** Daheng Wang, Zhiguang Guo, Weimin Liu

**Affiliations:** ^1^State Key Laboratory of Solid Lubrication, Lanzhou Institute of Chemical Physics, Chinese Academy of Sciences, Lanzhou, China; ^2^Hubei Collaborative Innovation Centre for Advanced Organic Chemical Materials and Ministry of Education Key Laboratory for the Green Preparation and Application of Functional Materials, Hubei University, Wuhan, China; ^3^University of Chinese Academy of Sciences, Beijing 100049, China

## Abstract

Inspired by nature's water-repellent plants, the superhydrophobic surface (SHS) and the lubricant-infused surface (LIS) possess potentials in various fields of application. In particular, the edible SHS and the edible LIS (ELIS) are suitable for the role of high-valued liquid food residue reduction. In this study, the ELIS was introduced through a facile spray method and direct lubricant infusion. Four types of ELISs were fabricated: carnauba wax with ethyl oleate infusion, carnauba wax with cooking oil infusion, beeswax with ethyl oleate infusion, and beeswax with cooking oil infusion. The carnauba wax-coated ELIS has better slipperiness, while the beeswax-coated ELIS has better transparency. The ethyl oleate-infused ELIS possesses ELIS to SHS transformable ability, and the cooking oil-infused ELIS also possesses better slipperiness and has the affordable advantage. Moreover, the material selection of ELIS is accessible, renewable, green, recyclable, and edible. The results illustrated that ELIS has advantages of long-term effectiveness and impact resistance over edible SHS and indicated that the ELIS can be facilitated for the manufacture of a multifunctional liquid residue reduction surface with food safety assurance.

## 1. Introduction

The remaining residue in the package of food after consumption has been a major issue with regard to the food wasting problem and hygiene issue. Commercial liquid food products are often packed with a container made from plastics; after consumption, liquid can adhere onto the bottle inner surface leading to food wasting. Data showed this phenomenon could lead to up to 15% of food waste, especially in the case of sticky and high-valued food such as yogurt [1–8]. Efforts for providing a residue reduction ability for a food-related surface have been made by scientists around the world. Edible superhydrophobic surfaces with self-cleaning ability were brought up to provide residue reduction [9–12]. Superhydrophobic surfaces inspired by nature's lotus plant leaf have huge potentials in the fields of anti-icing [13], antifouling [14], antifogging [15, 16], and antifrosting [17, 18]. Traditionally perfluorinated compounds (PFCs) and their derivatives were applied for the fabrication of superhydrophobic surfaces; however, those chemicals are potentially harmful to mammals and the environment [19–21]. Since superhydrophobic materials are applied for food-related surfaces, the material selection, extra precautions, and corresponding legal regulations should be taken into consideration. Therefore, carnauba wax and beeswax, two organic waxes classified by the U.S. Food and Drug Administration (FDA) as GRAS (generally recognized as safe; 21CRF184.1978 and 21CFR184.1973) were chosen as the raw materials of superhydrophobic surfaces [9, 10]. Moreover, the existing designs of a superhydrophobic surface could be fragile under circumstances of abrasion in which leaching of the coating is inevitable [22, 23].

The reasons that the traditional superhydrophobic surfaces were able to repel liquid are mainly due to the efforts of hydrophobic chemical compositions and the micro/nanostructures [24, 25]. When the superhydrophobic surface is in contact with liquid in an atmospheric environment, the air cushion trapped by micro/nanostructures and hydrophobic chemicals in contact with the liquid provided the surface with resistance. However, the air cushion can be breached; therefore, causing the surface to lose superhydrophobicity [26, 27]. From the perspective of safety and stability, an alternative option is a lubricant-infused surface (LIS), inspired by Nepenthes pitcher plants [28]. To address this stability issue, the lubricant introduced could endow the surface with long-term water resistance by replacing the air cushion with an oil film, therefore endowing the surface with a slippery property [29]. This acquired ELIS could achieve the equivalent liquid resistance ability of the edible superhydrophobic surface with long durability expectancy. Moreover, the lubricants endowed the surface with low hysteresis when in contact with liquids.

Additionally, all materials applied for the fabrication of ELIS are organic, renewable, edible, and biodegradable. In this work, an edible lubricant-infused surface (ELIS) was fabricated by infusing edible lubricants into a porous structure constituting edible waxes. Lubricants can fill the microstructures between wax particles and nanostructures on them. Four types of ELIS were prepared: carnauba wax-coated porous structure infused with ethyl oleate (EO), carnauba wax-coated porous structure infused with cooking oil (CO), beeswax-coated porous structure infused with EO, and beeswax-coated porous structure infused with CO. Both carnauba wax and beeswax are accessible and renewable resources. EO can be used as a flavoring agent for food additives; the usage of EO is regulated by food authorities. FDA regulation classified ethyl oleate as a food additive permitted for direct addition to food for human consumption. The World Health Organization (WHO) stated that the threshold for human intake of ethyl oleate is 1800 micrograms per day [30]. CO used in this study is soybean oil, extracted from soybean; CO has a long history for daily cooking; therefore, it is safe for human consumption [31]. Moreover, EO can dissolve in ethanol and endows the ELIS with facile transformation between a superhydrophobic material and the ELIS. Therefore, ELIS might have further potentials in the making of food packaging.

Here, a bioinspired multifunctional edible lubricant-infused slippery coating was prepared by a facile spraying method with simple direct lubricant infusion. The coating can be applied onto the substrate of glass and Polyethylene Terephthalate (PET). To evaluate the practicality of ELIS, the obtained ELIS samples were put to experiments on slippery analysis, liquid food residue reduction test, water jetting test, film bending test, icing delay analysis, and lubricant retention test.

## 2. Results and Discussion

### 2.1. Preparation of Edible Hydrophobic Porous Structure (HC) and ELISs and Characterization

**Figure 1:**
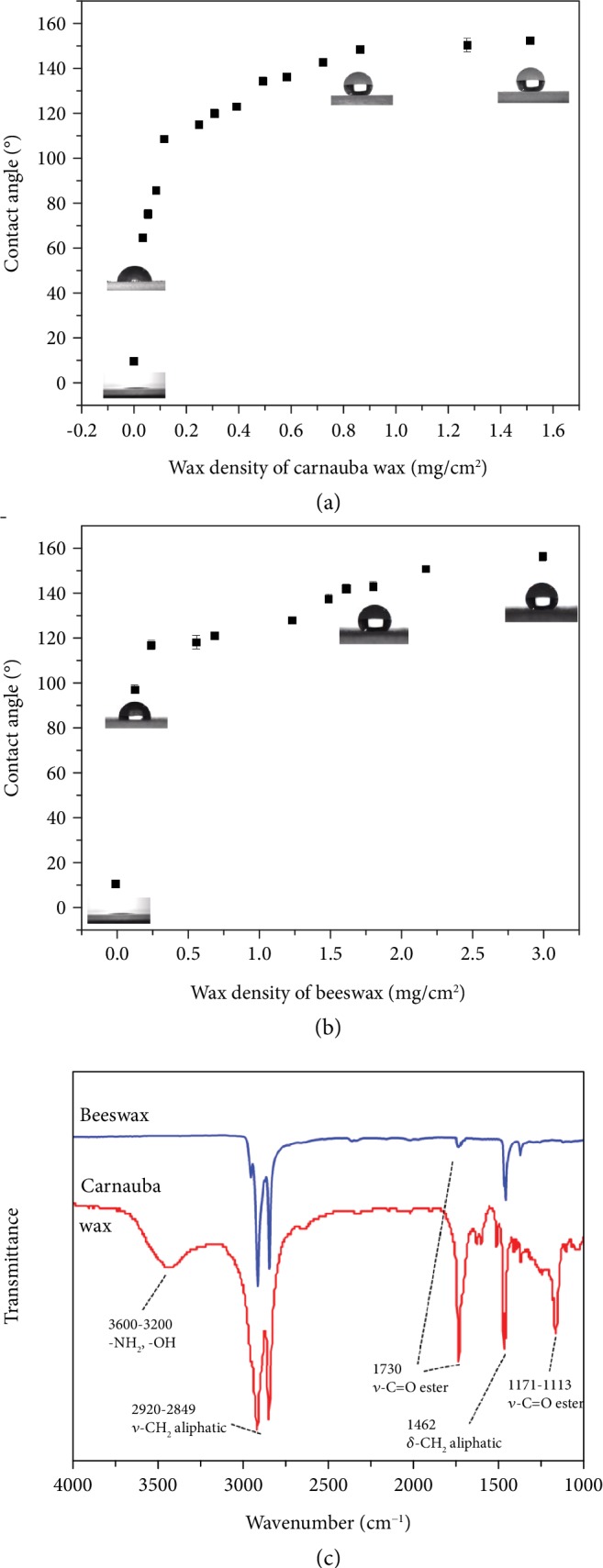
Wettability test of wax-coated SHS and FT-IR results of wax. (a, b) The relationship between carnauba wax density and the contact angle of deionized water on the coated surface. (c) The FT-IR result of carnauba wax and beeswax.

The edible HC surfaces were fabricated by spraying the wax-ethanol emulsion of carnauba wax and beeswax. The wax-ethanol emulsion was prepared by dissolving wax in ethanol at relatively high temperature and precipitate waxes at room temperature. The precipitated wax can form a smaller-sized particle, as both carnauba wax and beeswax appear to be soluble in high-temperature ethanol and insoluble in relatively cold ethanol. The reason that carnauba wax and beeswax were chosen as the raw materials for ELIS is the water repellence potential these two waxes have. Carnauba wax and beeswax can form superhydrophobic surfaces (SHS) via a spraying method [9]. As shown in Figures 1(a) and 1(b), the obtained spray-coated surface can gain superhydrophobicity (CA > 150°), when wax density reaches full coverage of the substrate (approximately 1.2 mg/cm^2^ for carnauba wax and 2.5 mg/cm^2^ for beeswax) [10]. Moreover, as displayed in Figure 1(c), the FT-IR chart indicated that both carnauba wax and beeswax possess the *ν*-CH2 aliphatic and *δ*-CH2 aliphatic groups at wavenumber vibration peaks of 2920 cm^−1^ to 2849 cm^−1^ and 1462 cm^−1^, respectively. The effect of hydrophobicity is mainly due to long-chain aliphatic groups [32]. The FT-IR result of carnauba wax at the wide peak around 3600 cm^−1^ to 3200 cm^−1^ reflected the stretching vibration of the hydroxyl and amino groups. The vibration peak at 1730 cm^−1^ can be attributed to the stretching vibration of the C=O ester group. The small vibration at 1171 cm^−1^ and the vibration peak of 1113 cm^−1^ represent the C-O ester group.

The carnauba wax possesses the hydroxyl and amino groups, which are chemical reactive groups that can form a hydrogen bond with others [33–35]. To address this issue, lubricant infusion could reduce the hydrogen bond formation by isolating the wax-constituted porous structure from a surface contaminant.

**Figure 2:**
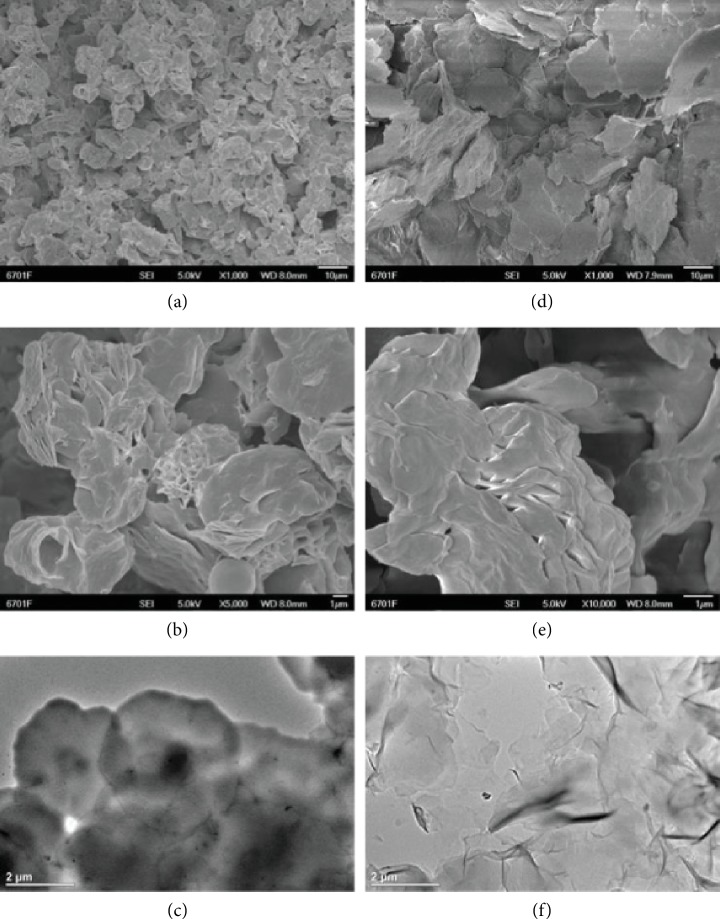
SEM and TEM results of the wax particles of coating. (a, b) The SEM images of carnauba wax coating structure. (c) The TEM image of carnauba wax particles. (d, e) The SEM images of beeswax coating structure. (f) The TEM image of beeswax particles.

To explore the microstructure of the coated waxes and seek feasibility of the lubricant retention, the images for various scales of the wax coatings and wax particle were captured by the SEM and TEM. The SEM images of the coated surface with carnauba wax and beeswax densities of full surface coverage (1.2 mg/cm^2^ and 2.5 mg/cm^2^ separately) were captured. As shown in Figures 2(a) and 2(c), the enlarged images indicate that lump-like particles of carnauba wax and flake-like particles of beeswax stacked with other particles and covered the coating substrate. These stacked dense structures endow the coated surface with superhydrophobicity; moreover, the gaps in between stacked particles provide the space for lubricant infusion. As shown in Figures 2(b) and 2(e), the two-micrometer scale images clearly show that there are nanoscale porous structures on top of the lump-like particles of carnauba wax. Moreover, there are nanoscale porous structures existing in between layers of flake-like beeswax particles. The nanoscale porous structures existed on both waxes' particles which further provided superhydrophobicity and space of lubricant infusion for the coated surface [36, 37]. As shown in Figures 2(c) and 2(f), the TEM images of separated waxes' particles further examined the structures of carnauba wax and beeswax particles. The lump-like particles of carnauba wax are scaled at approximately two micrometers in diameter; moreover, the porous structure can be identified on top of the lump. The flake-like beeswax particles are also scaled at approximately two micrometers in diameter. Moreover, the flakes are wrinkled, which explains the formation of porous structures existing in between layers of flake-like beeswax particles.

**Figure 3:**
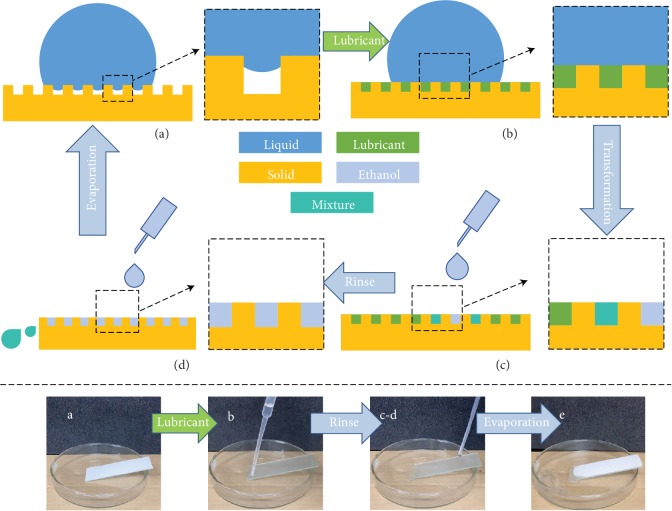
Schematic illustration of surface transformation and captured images of each stage. (a, b) The liquid contact state of HC and ELIS. (a–e) The schematic diagram of the transformation cycle between HC and ELIS (e) the recovered HC surface after evaporation (the mixture refers to ethanol with lubricant dissolved in).

The gaps in between stacked particles and the porous structures exist in between or on top of wax particles which provided the space for the lubricant infusion. Moreover, due to capillary phenomenon of the aforementioned micro/nanostructure, as illustrated in Figures 3(a) and 3(b), the lubricant can be smoothly absorbed by the wax-coated surfaces; therefore, the ELIS can achieve total replacement of air cushion with a lubricant film and endow the surface with low hysteresis ([Supplementary-material supplementary-material-1]). Furthermore, to achieve ELIS to SHS transformation, ethanol rinsing is a simple and quick passway, as lubricant ethyl oleate can quickly dissolve in ethanol at room temperature, while both waxes cannot (Figures 3(c) and 3(d) and [Supplementary-material supplementary-material-1]).

**Figure 4:**
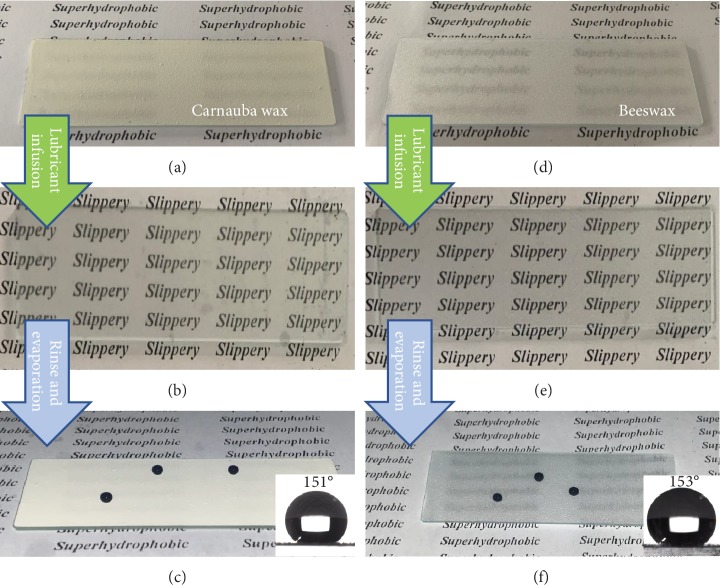
Illustration of transparency changes and wettability results, after state change. (a, d) Pictures of carnauba wax and beeswax SHSs. (b, e) ELISs after lubricant infusion. (c, f) SHSs restored from ELISs.

Moreover, the surface gained transparency after the lubricant infusion. The reason to that is the infusion-replaced low-density air with a higher-density lubricant, therefore altering the reflective index of the glass slides. As can be seen in Figures 4(b) and 4(e), occurrences of the word “Slippery” are visually clear when beneath both ELISs, while occurrences of the word “Superhydrophobic” are visually unreadable under the carnauba wax-coated SHS glass slide and visually blurred under the beeswax-coated SHS glass slide (Figures 4(a) and 4(d)). Moreover, the carnauba wax is visually white with slight yellow color and the yellow color of the carnauba wax has affected the color of the carnauba wax ELIS, while the beeswax-coated glass slide is visually white and translucent. The transparency ELIS has gained could be beneficial to the ELIS-coated packaging with quality and volume visibility of the contained content. Furthermore, through ethanol rinsing, the HC surface or SHS can be recovered from the ELIS with the hydrophobicity uncompromised (Figures 4(c) and 4(f)). Furthermore, the lubricant infused in ELIS such as EO can dissolve in ethanol leading to slippery loss of the ELIS when against alcohol drinks like wine and beer. However, ELIS and SHS transformation can endow a modified surface with residue reduction ability for a wider range of liquids [10]. Furthermore, due to the reason that all materials used for ELIS fabrication can be dissolved in high-temperature organic solvents such as ethanol, the recycling of the ELIS-coated substrate can be possible.

**Figure 5:**
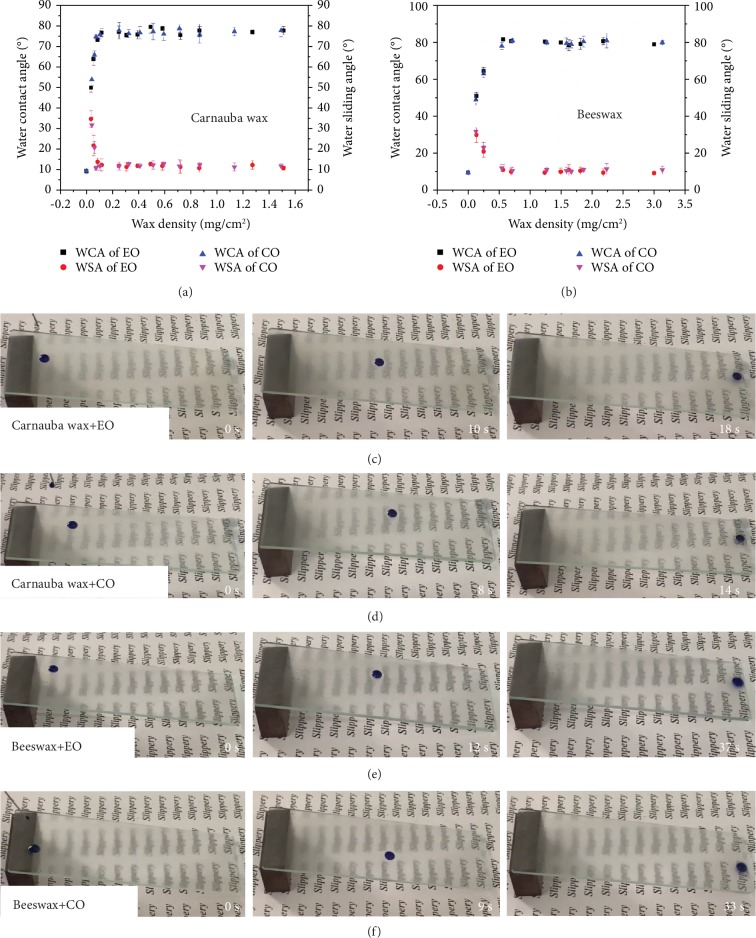
Wettability and slipperiness results of ELISs. (a) The relationship between CA, SA, and wax density of carnauba wax. (b) The relationship between CA, SA, and wax density of beeswax. (c–f) The self-slippery experiment of four different ELISs. (Water was dyed with methyl blue.)

The slippery test was conducted in two directions: the water contact angle and the sliding angle on the ELIS and the sliding speed of water droplet on a tilted ELIS glass. As can be seen in Figures 5(a) and 5(b), the water contact angle (CA) and the water sliding angle (SA) of ELIS increase as the wax density gets higher, until the wax density reaches the threshold (approximately 0.1 mg/cm^2^ for carnauba wax and 0.5 for mg/cm^2^ for beeswax), then the CA and SA were maintained at a certain level (approximately 80° for CA and 10° for SA). The sliding motion of five microliter droplets on ELISs was also recorded (Figure 5(c)–5(f)). The start, midpoint, and end of the droplet sliding show that droplets on carnauba wax-coated ELISs possessed faster sliding speed than the beeswax-coated ones (18 s against 37 s and 14 s against 33 s). Moreover, the droplets on CO-infused ELISs hold slightly faster sliding speed against EO-infused ones (14 s against 18 s and 33 s against 37 s). The speed disparities between two wax-coated ELISs resulted from the morphology difference of the ELISs. As mentioned before, the beeswax particles are flake like, which lead to uneven micromorphology, further affecting the smoothness of the surface. In comparison, the lump-like carnauba wax particles stacked and formed a smoother surface, due to the vertical performing nature of the spray method. The surface morphology measurement indicated that the surface roughness of carnauba wax coating (Ra = 0.0293 ± 0.005 *μ*m) is lower than that of the beeswax coating (Ra = 1.39 ± 0.15 *μ*m) ([Supplementary-material supplementary-material-1]). In comparison, carnauba wax with the CO ELIS holds better water slippery performance when against others.

### 2.2. Liquid Food Residue Reduction Experiments

**Figure 6:**
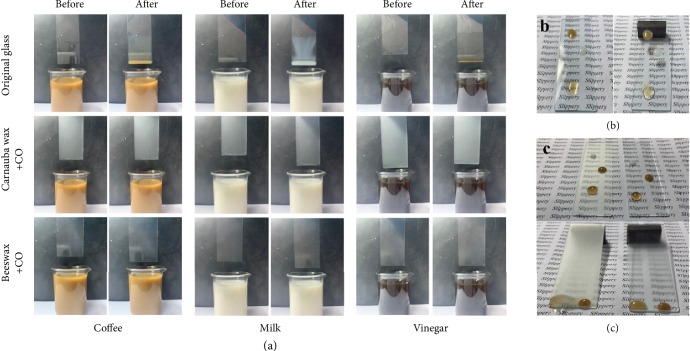
Stickiness test of ELISs against various liquid foods. (a) Liquid food impregnation of CO-infused carnauba wax and beeswax ELISs (control: original glass). (b) Liquid food (coffee, milk, vinegar from top to bottom) contact state on a horizontal flat original glass slide and a tilted (approximately 10°) original glass slide. (c) Liquid food (milk, coffee, and vinegar from top to bottom) contact state on horizontal flat CO-infused carnauba wax and beeswax ELISs and tilted ones.

To further explore the slippery performance against various liquid foods, two types of wax-coated SHS and two types of CO-infused ELIS were chosen as test subjects for a food liquid impregnation test. As shown in [Supplementary-material supplementary-material-1], both sides of wax-coated glasses showed antifouling ability due to the superhydrophobicity of the wax coating. As can be seen in Figure 6(a), after fifty times of impregnation of glasses with ELIS coated on both sides, the surfaces of the original glasses have been attached with all three types of liquid food. On the contrary, both the carnauba wax and beeswax with CO-infused ELISs did not retain any liquid food residue and have no visual changes to the appearance, which indicated that the ELIS could achieve the equivalent liquid resistance ability of the edible superhydrophobic surface. As for the slippery test for tilted surfaces (Figures 6(b) and 6(c)), the food liquid remained adhere on the original glass after the glass slide been tilted.

**Figure 7:**
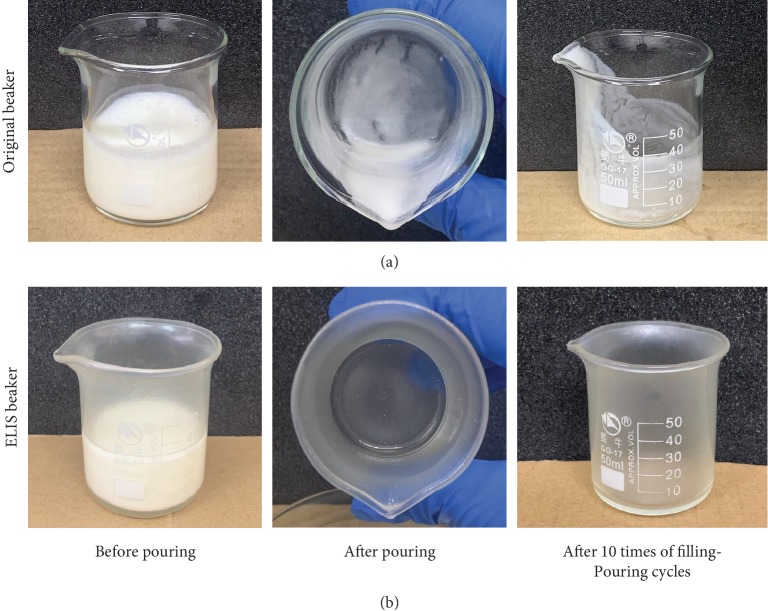
Liquid food residue reduction illustration of ELIS coating when against yogurt. (a, b) Pictures of the untreated beaker and the ELIS beaker (filled with 30 ml yogurt) before pouring, after pouring, and after 10 times of filling-pouring cycles.

A further practical experiment was applied to test ELIS. A practical mode for liquid food reduction was built: the inner wall of the beaker (50 ml) was coated with carnauba wax with the CO-infused ELIS. Various liquid foods were chosen as the subject of a filling and pouring test for the untreated and treated beakers. Liquid foods include coffee, milk, vinegar, coke, green tea, energy drink, and yogurt. The most consumed commercial liquid food products were selected; moreover, the selection represented different types of foods: water-fat emulsion-based drink, alkali-based drink, acid-based drink, sticky food, carbonated drink, salty drink, and bacteria-based products. The ELIS-coated beaker showed great residue reduction ability to all test subjects (Table 1). In particular, for sticky liquid such as yogurt, the residue reduction rate is significant, even after ten times of filling-pouring cycles (Figures 7(a) and 7(b); [Supplementary-material supplementary-material-1]-[Supplementary-material supplementary-material-1]). The outcome correlates with the results in the impregnation test. Results of both tests indicate that the ELIS holds potential for liquid food residue reduction.

### 2.3. Durability and Lubricant Retention Tests

**Figure 8:**
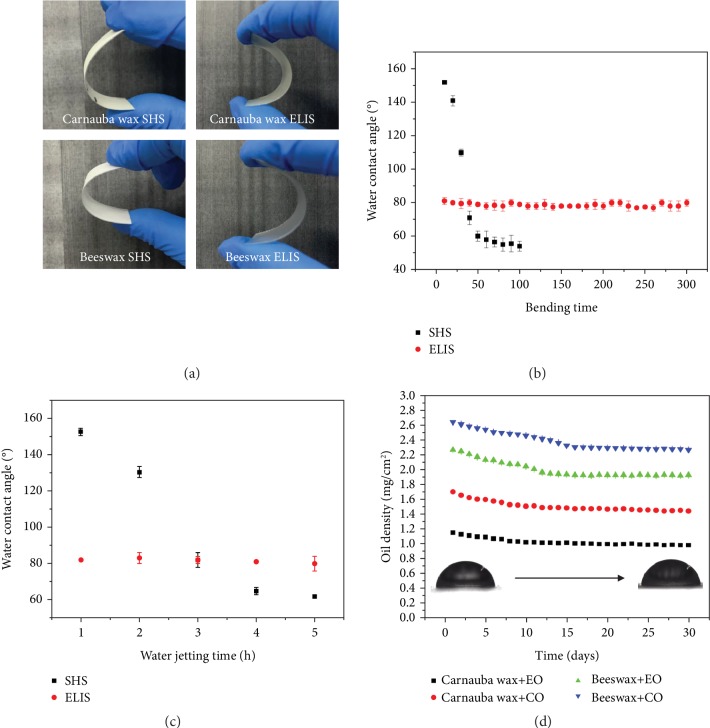
Results of durability test and lubricant retention tests of ELIS coating. (a) The bending illustration of carnauba wax-coated SHS and CO-infused ELIS (the coated pet film was bent 180°). (b) Bending durability of SHP and ELIS. (c) Water jetting durability of SHP and ELIS. (d) Lubricant retention ability tests for 30 days.

To test the durability of the ELIS as the coating for the liquid food packaging inner wall, the water jetting test and bending test were conducted. These two tests are to mimic the impact to the inner wall of commercial packaging. Four types of the ELIS-coated PET film were bent 100 times each (Figure 8(a)). For the SHS (carnauba wax coated), the water contact angle declined as the bending time increased (Figure 8(b)). On the other hand, the ELIS can resist the impact of bending, as the water contact angle had minor change after 300 times of bending (Figure 8(b)). Moreover, the water contact angle of the SHS dropped significantly as the SHS sample suffered from the bending and loss of wax particles, while the ELIS sample (carnauba wax coated with CO infusion) remained its primary condition ([Supplementary-material supplementary-material-1]). Therefore, the ELIS can bear extensive hours of water jetting, while the SHS cannot (Figure 8(c)). Both the water jetting and bending test proved that the lubricant-infused ELIS holds stability advantage over the SHS. The additional stability of the ELIS is provided by the introduced lubricant. By comparing the SHS with the ELIS, the infusion of lubricant in the ELIS replaced the air in the porous structure of the SHS with relatively higher-density long-chain lubricants, which provided additional van der Waals force [38]. Therefore, the infusion increased the solidarity and transparency of the ELIS; furthermore, it strengthened the binding force between the substrate and ELIS coating. To further test the long-term durability of the ELIS, the lubricant retention test was also conducted. As illustrated in Figure 8(d), all ELISs exhibited strong retention ability for infused lubricants. The loss of lubricant occurred at the initial stage of the water immersion. Due to the reason that densities of both EO and CO are lower than that of water. However, the lubricant retention rate maintained at around 85% after the initial stage. Furthermore, after 30 days of water immersion, the CA remained at around 80° ([Supplementary-material supplementary-material-1]). This result indicated that the long-term lubricant retention abilities of all ELISs are remarkable. For the purposes of food packaging application, the stability and long-term effectiveness of the ELIS are essential. The shelf life of liquid food product such as coke and energy drinks is around 12 months; therefore, the ELIS could be suitable for the role of additional packaging material with liquid reduction ability.

### 2.4. Food Safety Discussion

To determine whether the food safety standard of the ELIS food packaging fits the regulations of authorities, the daily intake of lubricant was also calculated. Taking carnauba wax with the CO-infused ELIS, for example, the maximum lubricant loss is around 0.1885 mg/cm^2^ ([Supplementary-material supplementary-material-1]). The contact area of cylindrical food container (*S*) can be calculated as follows:

(1) $$\begin{array}{c} S=2\pi r\times \frac{V}{\pi r^{2}}+\pi r^{2}. \end{array}$$

*V* and *r* represent the volume and bottom radius, respectively, of the cylindrical food container. Taking yogurt product, for example, the United States Department of Agriculture (USDA) recommends three cups of yogurt per day for an adult human (around 200 ml per cup with bottom radius of 4 cm). Therefore, the contact area (*S*) can be calculated through equation (1) as 150.24 cm^2^; moreover, the maximum daily intake of lubricant in yogurt product can be calculated as 85 mg, which is well below the threshold of 1800 mg as mentioned before. Therefore, the application of ELIS is well fitting the food safety regulations of food authorities.

### 2.5. Icing Delay Experiment

**Figure 9:**
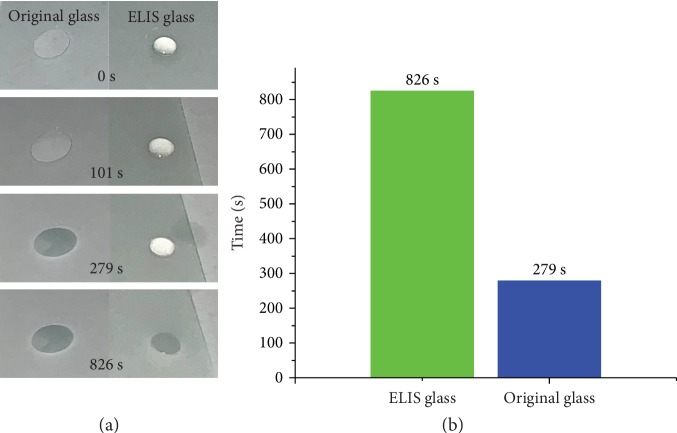
Icing delay experiment results. (a) Recorded ice acumination on the original glass (left) and carnauba wax with the CO ELIS glass (right) at -5°C. (b) Icing time comparison of ELIS and original glasses.

The anti-icing property of a surface has profound meaning for the outdoor equipment and systems. In the case of food packaging surfaces, the prohibition of ice acumination is also meaningful. For carbonated drinks, icing could lead to the fast release of carbon dioxide, due to the reason that solidification of water decreases the dissolution rate of carbon dioxide and causes the flavor loss of the product. Moreover, the release of carbon dioxide can increase air pressure of the package, leading to potential hazards and economic losses. Other than that, for containers with high brittleness such as the glass bottle, the icing of the inner content can lead to breach or destruction of the container due to the swelling of the icing content. The introduction of ELIS to packaging materials such as glass could reduce the ice accumulation by reducing the pinning spot for ice nucleation [39, 40]. The carnauba wax with CO was chosen as a test surface due to better slippery performance of the coating and lower icing point of CO (approximately -20°C). As shown in Figure 9(a), recordings conformed the icing delay effect of ELIS. The water droplet on the original glass remained transparent until 279 s, and the droplet on the ELIS glass turned into opaque ice at 826 s. Moreover, as illustrated in Figure 9(b), the icing time of the droplet on the ELIS glass is significantly longer, which correlates with the recordings and proves the icing delay ability of the ELIS-coated glass.

## 3. Conclusion

The edible superhydrophobic surfaces have been reported in previous researches [9–12, 22]. However, there still lack long-term durability, recyclable property, and transparency for previous surfaces to improve. Therefore, the introduced edible carnauba wax- and beeswax-coated surfaces with ethyl oleate and cooking oil infusion ELIS can be an alternative option for high-valued liquid food residue reduction and icing delay packaging design. To compensate for the insufficient alcohol-based liquid repellency of ELIS, the transformable passway between ELIS and SHS has also been reported in this study. Moreover, the various test results showed that the ELIS possesses long-term lubricant retention ability, impact resistance, and icing delay ability and excellent liquid food residue reduction ability against different types of liquid food. Therefore, the ELIS fabricated in this study could provide guidelines for further ELIS designs with other lubricants and coating materials. Moreover, this study provides the industry with economical multifunction packaging material with recyclable abilities.

## 4. Experimental

### 4.1. Materials

Carnauba wax was purchased from Shanghai Macklin Biochemical Co. Ltd., China. Beeswax was purchased from Solarbio Science & Technology Co. Ltd., China. Ethyl oleate was purchased from TCI, China. Cooking oil was purchased from Jinlongyu, China. The spray gun was supplied by Lotus Spray Gun, China. The glass slide and PET film were purchased from a commercial store. Ethanol was purchased from Rionlon, China. Deionized water was purified by the ModuPure system. All chemicals are AR grade.

### 4.2. Preparation of Edible Hydrophobic Porous Structure (HC)

First, two grams of carnauba wax and that of beeswax were added into a flask containing two hundred milliliters of ethanol, separately. Then, the mixtures were heated for two hours for the waxes to dissolve: 85°C for carnauba wax and 65°C for beeswax. Then, the solutions were cooled to room temperature till the wax contents were fully precipitated. After precipitation, the emulsions were then treated with ultrasonication for two hours; then the wax-ethanol emulsions for spray were obtained. The spray emulsions were then loaded into the spray gun with the spray pressure set at around twenty psi. During spray, the nozzle of the spray gun was held at a distance around twenty centimeters from the spray substrate (glass slide and PET film).

### 4.3. Transformation between HC Surface and ELIS

The coated substrates (glass slide and PET film) were set tilted at a certain angle. Then, the lubricants (ethyl oleate and cooking oil) were infused into the surface from the upper end of the coated substrates drop by drop. After the infusion, the excess lubricants were removed by rinsing with deionized water; then, the ELIS was obtained. To restore the HC surface from the ELIS, apply ethanol to EO-coated ELISs by jetting, then let ethanol evaporate at room temperature.

### 4.4. Characterization

The photographs and videos were taken by using smartphones. The images of the wax structures were captured using the field emission scanning electron microscope (FESEM, JSM-6701F) before Au-sputter. Images of wax particles were obtained on a transmission electronic microscope (TEM, FEI Tecnai G2 F30). The surface morphology of the samples was measured by a noncontact 3D surface profiler (MicroXAM-800). The chemical composition of the specimens was analyzed by Fourier transform infrared spectroscopy (FT-IR, Thermo Scientific Nicolet iS10). The water contact angle (WCA) was measured by using the JC2000D system (Zhong Chen Digital Equipment Co. Ltd., Shanghai, China) with deionized water (5 *μ*l) at room temperature. The sliding angle (SA) was measured by using the DSA100 contact angle meter. The WCA and SA readings of each samples were measured three times and averaged for result.

### 4.5. Liquid Food Residue Reduction Experiment

The original glass, edible SHS, and ELIS were immersed in milk, vinegar, and coffee; the cleanliness of these surfaces was observed after being immersed for thirty times. The same food liquids were dropped onto the sloped ELIS, and the sliding of liquid droplets was observed and captured.

### 4.6. Durability Tests

The durability tests of the edible HC and ELIS were carried out by two separate experiments: (1) The mechanical stability of the edible HC and ELIS was analyzed by water jetting and film bending tests. (2) The oil retention ability of the ELIS was reviewed by soaking the specimen into deionized water for thirty days; weight changes were recorded with a time interval of twenty-four hours.

### 4.7. Icing Delay Experiment

The icing delay experiment was conducted by placing the ELIS under the temperature condition of -5°C. Droplets of deionized water (5 *μ*l) were placed on the surface before cooling. After the cooling started, the freezing time of the droplets on each sample was recorded and compared.

## Figures and Tables

**Table 1:** Liquid food residue reduction results of beakers when against various liquid foods. The residue test to selective foods (30 ml) for untreated and ELIS-treated glass beakers (carnauba wax coating with CO infusion) and the reduction rate of each liquid food after comparison.

Liquid foods (50 ml)	Residue amount of original beaker (g)	Residue amount of ELIS beaker (g)	Liquid food residue reduction rate of ELIS
Coffee	0.48 ± 0.02	0.0054 ± 0.03	98.88%
Milk	0.9995 ± 0.09	0.2673 ± 0.2	73.26%
Vinegar	0.7290 ± 0.03	0.1501 ± 0.02	79.42%
Coke	1.203 ± 0.04	0.1321 ± 0.04	98.97%
Green tea	1.0072 ± 0.03	0.0103 ± 0.02	98.98%
Energy drink	1.139 ± 0.02	0.0114 ± 0.01	98.99%
Yogurt	2.9282 ± 0.08	0.1341 ± 0.09	95.42%

## Supplementary Materials

Supplementary Materials Table S1: the lubricant retention performance for all four types of ELIS. As calculated, the lubricant retention rate is around 85%. Table S2: the lubricant retention performance of carnauba wax-coated ELIS with CO infusion in yogurt. As calculated, the lubricant retention rate is 89.30%. The storage condition was set as 5°C for 7 days. Figure S1: advancing and receding contact angle of ELISs with CO infusion. 5 *μ*l distilled water was placed on the tilted carnauba wax- and beeswax-coated ELISs. Figure S2: surface morphology and roughness measurement. As measured through a noncontact 3D surface profiler, the surface roughness of carnauba wax (Ra = 0.0293 ± 0.005 *μ*m) is lower than the surface roughness of beeswax (Ra = 1.39 ± 0.15 *μ*m). Figure S3: stickiness test for SHS. Liquid food impregnation of carnauba wax and beeswax SHS in coffee, milk, and vinegar. Figure S4: the after-bend condition of SHS and ELIS coatings. The water contact angle of SHS dropped significantly as the SHS sample suffered from the bending and loss of wax particles, while the ELIS sample retained its primary condition. Movie S1: the SHS-ELIS-SHS transformation process was recorded. From the beginning, as the lubricant was dipped onto the SHS, the color of the surface turned into a transparent ELIS and the surface became slippery. Afterwards, the ELIS was rinsed with ethanol and heated for the surface to return to the SHS state. Movie S2: the pouring test of untreated beakers was conducted against yogurt. Movie S3: the pouring test of ELIS-treated beakers was conducted against yogurt. Movie S4: the pouring test of ELIS-treated beakers was conducted against coffee. Movie S5: the pouring test of ELIS-treated beakers was conducted against green tea.
