# Supplementary material for: Bioinspired Edible Lubricant-Infused Surface with Liquid Residue Reduction Properties
Source: Research (Wash D C). 2019 Oct 10;2019:1649427. doi: 10.34133/2019/1649427 (PMC6946289; doi:10.34133/2019/1649427)
Supplement: Supplementary Materials — Table S1: the lubricant retention performance for all four types of ELIS. As calculated, the lubricant retention rate is around 85%. Table S2: the lubricant retention performance of carnauba wax-coated ELIS with CO infusion in yogurt. As calculated, the lubricant retention rate is 89.30%. The storage condition was set as 5°C for 7 days. Figure S1: advancing and receding contact angle of ELISs with CO infusion. 5 μl distilled water was placed on the tilted carnauba wax- and beeswax-coated ELISs. Figure S2: surface morphology and roughness measurement. As measured through a noncontact 3D surface profiler, the surface roughness of carnauba wax (Ra = 0.0293 ± 0.005 μm) is lower than the surface roughness of beeswax (Ra = 1.39 ± 0.15 μm). Figure S3: stickiness test for SHS. Liquid food impregnation of carnauba wax and beeswax SHS in coffee, milk, and vinegar. Figure S4: the after-bend condition of SHS and ELIS coatings. The water contact angle of SHS dropped significantly as the SHS sample suffered from the bending and loss of wax particles, while the ELIS sample retained its primary condition. Movie S1: the SHS-ELIS-SHS transformation process was recorded. From the beginning, as the lubricant was dipped onto the SHS, the color of the surface turned into a transparent ELIS and the surface became slippery. Afterwards, the ELIS was rinsed with ethanol and heated for the surface to return to the SHS state. Movie S2: the pouring test of untreated beakers was conducted against yogurt. Movie S3: the pouring test of ELIS-treated beakers was conducted against yogurt. Movie S4: the pouring test of ELIS-treated beakers was conducted against coffee. Movie S5: the pouring test of ELIS-treated beakers was conducted against green tea. [file 1649427.f1.zip › SI revised.docx]

**Supplementary Materials**

**Table. S1: The lubricant retention performance for all four types of ELIS.** As calculated the lubricant retention rate is around 85%.

| days/times | Lubricant weight (mg/cm^2^) | | | |
| --- | --- | --- | --- | --- |
|  | beeswax EO | beeswax CO | carnauba wax EO | carnauba wax CO |
| 1 | 2.272 | 2.645 | 1.152 | 1.707 |
| 2 | 2.251 | 2.619 | 1.131 | 1.659 |
| 3 | 2.213 | 2.587 | 1.115 | 1.627 |
| 4 | 2.176 | 2.565 | 1.099 | 1.605 |
| 5 | 2.139 | 2.544 | 1.093 | 1.600 |
| 6 | 2.133 | 2.512 | 1.072 | 1.579 |
| 7 | 2.101 | 2.501 | 1.067 | 1.563 |
| 8 | 2.080 | 2.491 | 1.040 | 1.531 |
| 9 | 2.075 | 2.480 | 1.035 | 1.525 |
| 10 | 2.048 | 2.464 | 1.024 | 1.509 |
| 11 | 2.011 | 2.443 | 1.024 | 1.515 |
| 12 | 1.968 | 2.421 | 1.019 | 1.493 |
| 13 | 1.952 | 2.400 | 1.019 | 1.493 |
| 14 | 1.947 | 2.368 | 1.013 | 1.493 |
| 15 | 1.941 | 2.331 | 1.019 | 1.488 |
| 16 | 1.931 | 2.309 | 1.008 | 1.477 |
| 17 | 1.931 | 2.307 | 1.008 | 1.483 |
| 18 | 1.925 | 2.304 | 1.003 | 1.477 |
| 19 | 1.936 | 2.301 | 0.997 | 1.483 |
| 20 | 1.931 | 2.299 | 0.997 | 1.472 |
| 21 | 1.931 | 2.296 | 0.992 | 1.472 |
| 22 | 1.931 | 2.293 | 0.997 | 1.477 |
| 23 | 1.925 | 2.291 | 1.003 | 1.467 |
| 24 | 1.936 | 2.288 | 0.992 | 1.461 |
| 25 | 1.931 | 2.285 | 0.987 | 1.461 |
| 26 | 1.925 | 2.283 | 0.992 | 1.456 |
| 27 | 1.936 | 2.288 | 0.981 | 1.445 |
| 28 | 1.931 | 2.283 | 0.987 | 1.451 |
| 29 | 1.925 | 2.283 | 0.981 | 1.456 |
| 30 | 1.931 | 2.272 | 0.981 | 1.445 |
| retention rate (%) | 84.99 | 85.90 | 85.15 | 84.65 |

**Table S2: The lubricant retention performance of carnauba wax coated ELIS with CO infusion in yogurt.** As calculated the lubricant retention rate is 89.30%. The storage condition was set as 5°C for 7 days.

| Days/Times | Lubricant weight (mg/cm^2^) |
| --- | --- |
| 1 | 1.7624 |
| 2 | 1.7512 |
| 3 | 1.7277 |
| 4 | 1.6516 |
| 5 | 1.6345 |
| 6 | 1.6321 |
| 7 | 1.5739 |
| retention rate (%) | 89.30 |

**
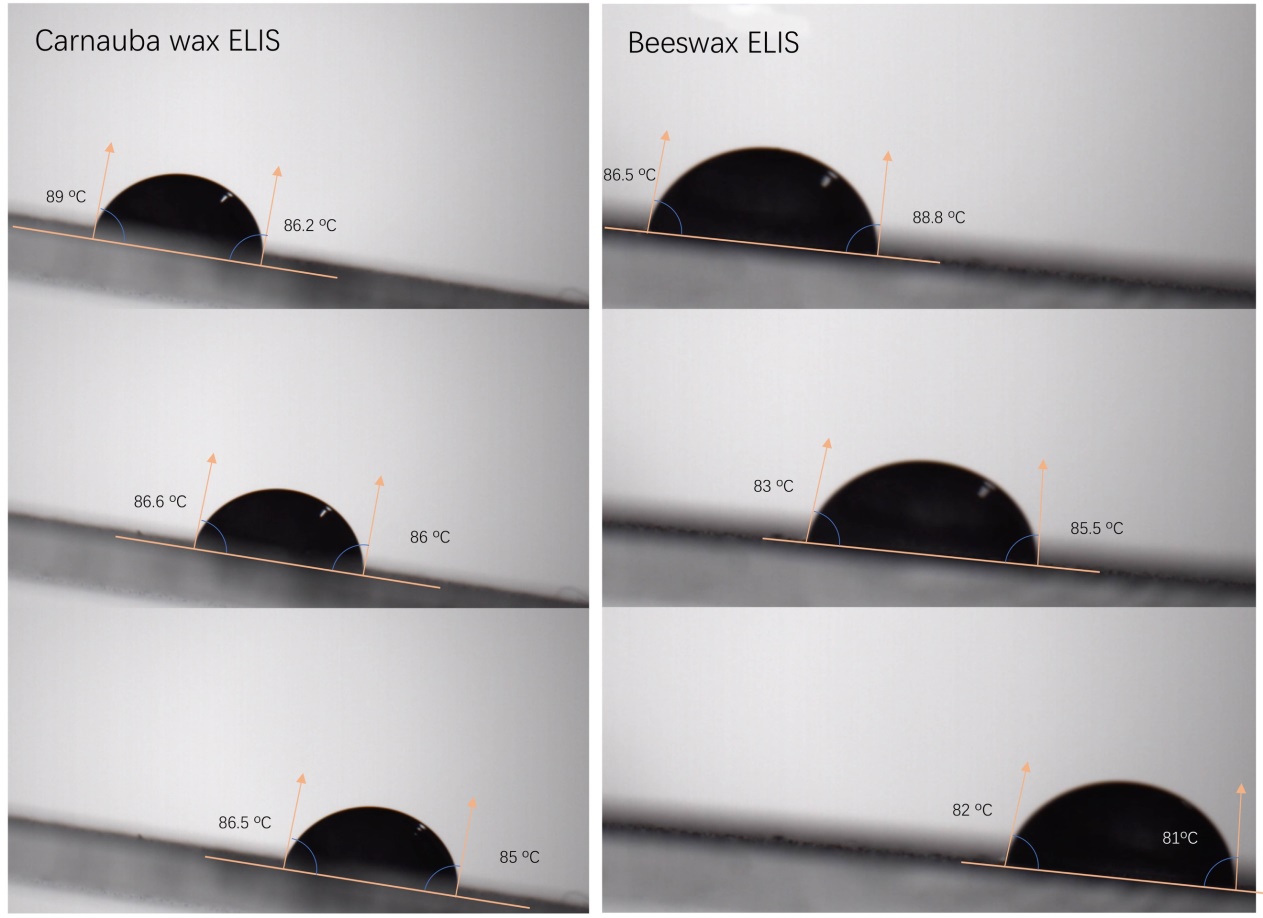
**

**Figure S1: Advancing and receding contact angle of ELISs with CO infusion.** 5 µl distilled water were placed on the tilted Carnauba wax ELIS and Beeswax ELIS coated surfaces.


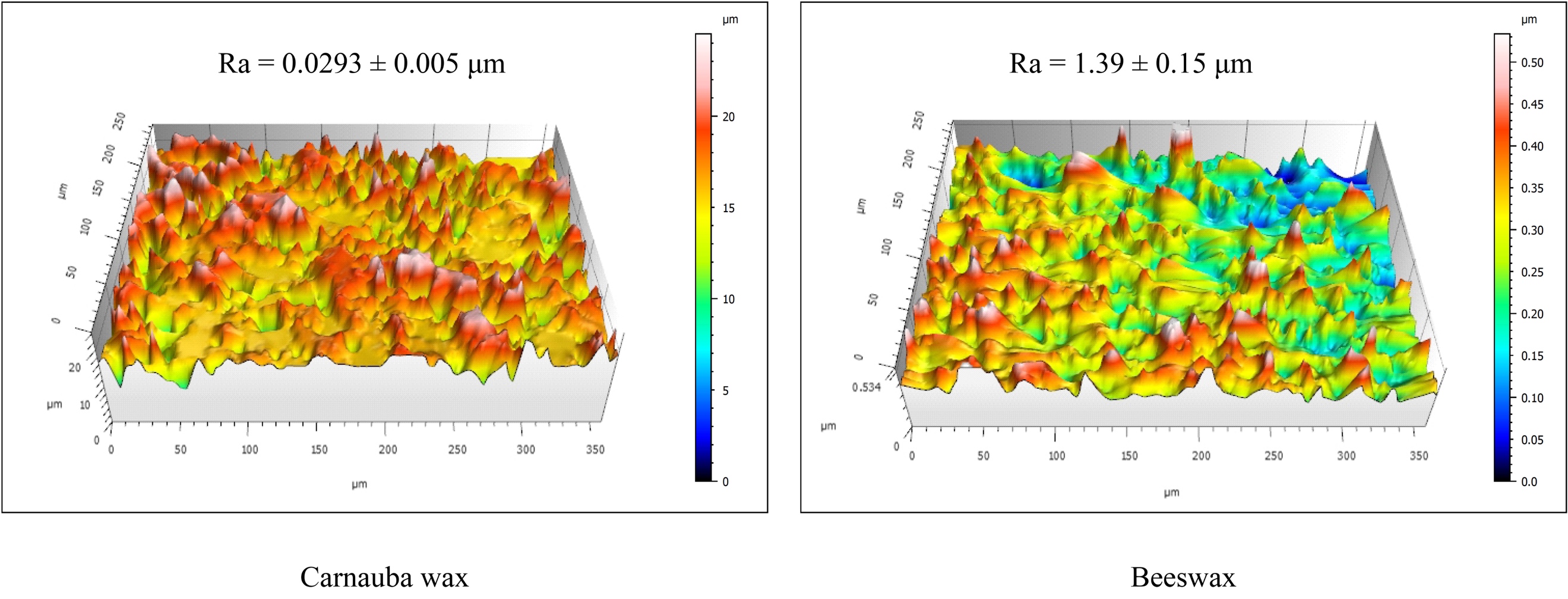


**Figure S2: Surface morphology and roughness measurement.** As measured through non-contact 3D surface profiler, the surface roughness of carnauba wax (Ra = 0.0293 ± 0.005 μm) is lower than the surface roughness of beeswax (Ra = 1.39 ± 0.15 μm).


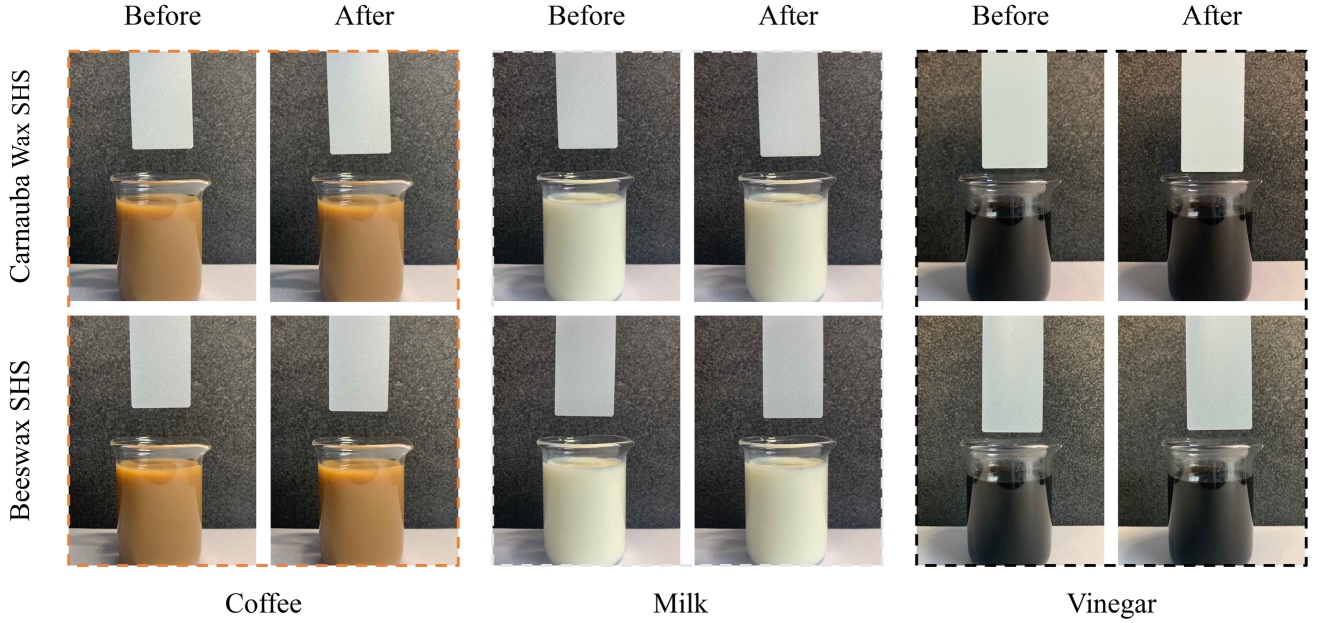


**Figure S3: Stickiness test for SHS surfaces.** Liquid food impregnation of carnauba wax and beeswax SHS in coffee, milk and vinegar.


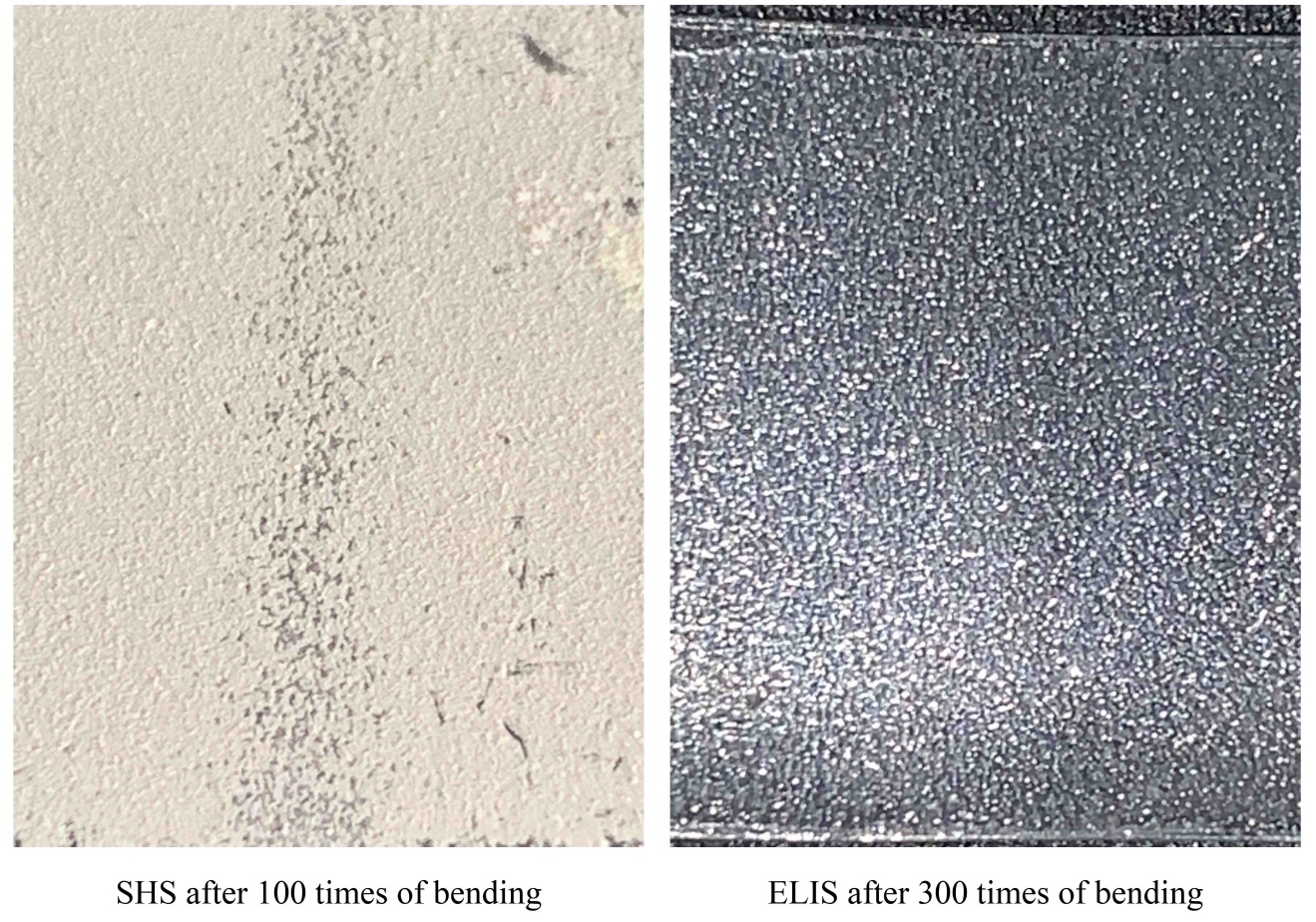


Figure S4: The after-bend condition of SHS and ELIS coatings. The water contact angle of SHS dropped significantly as the SHS sample suffered from the bending and loss of wax particles, while the ELIS sample remained its primary condition.
